# Iron is a centrally bound cofactor of specifier proteins involved in glucosinolate breakdown

**DOI:** 10.1371/journal.pone.0205755

**Published:** 2018-11-05

**Authors:** Anita Backenköhler, Daniela Eisenschmidt, Nicola Schneegans, Matthias Strieker, Wolfgang Brandt, Ute Wittstock

**Affiliations:** 1 Institute of Pharmaceutical Biology, Technische Universität Braunschweig, Braunschweig, Germany; 2 Department of Bioorganic Chemistry, Leibniz Institute of Plant Biochemistry, Halle (Saale), Germany; University of Illinois at Urbana-Champaign, UNITED STATES

## Abstract

Glucosinolates, a group of sulfur-rich thioglucosides found in plants of the order Brassicales, have attracted a lot of interest as chemical defenses of plants and health promoting substances in human diet. They are accumulated separately from their hydrolyzing enzymes, myrosinases, within the intact plant, but undergo myrosinase-catalyzed hydrolysis upon tissue disruption. This results in various biologically active products, e.g. isothiocyanates, simple nitriles, epithionitriles, and organic thiocyanates. While formation of isothiocyanates proceeds by a spontaneous rearrangement of the glucosinolate aglucone, aglucone conversion to the other products involves specifier proteins under physiological conditions. Specifier proteins appear to act with high specificity, but their exact roles and the structural bases of their specificity are presently unknown. Previous research identified the motif EXXXDXXXH as potential iron binding site required for activity, but crystal structures of recombinant specifier proteins lacked the iron cofactor. Here, we provide experimental evidence for the presence of iron (most likely Fe^2+^) in purified recombinant thiocyanate-forming protein from *Thlaspi arvense* (TaTFP) using a Ferene S-based photometric assay as well as Inductively Coupled Plasma-Mass Spectrometry. Iron binding and activity depend on E266, D270, and H274 suggesting a direct interaction of Fe^2+^ with these residues. Furthermore, we demonstrate presence of iron in epithiospecifier protein and nitrile-specifier protein 3 from *Arabidopsis thaliana* (AtESP and AtNSP3). We also present a homology model of AtNSP3. In agreement with this model, iron binding and activity of AtNSP3 depend on E386, D390, and H394. The homology model further suggests that the active site of AtNSP3 imposes fewer restrictions to the glucosinolate aglucone conformation than that of TaTFP and AtESP due to its larger size. This may explain why AtNSP3 does not support epithionitrile or thiocyanate formation, which likely requires exact positioning of the aglucone thiolate relative to the side chain.

## Introduction

**Fig 1:**
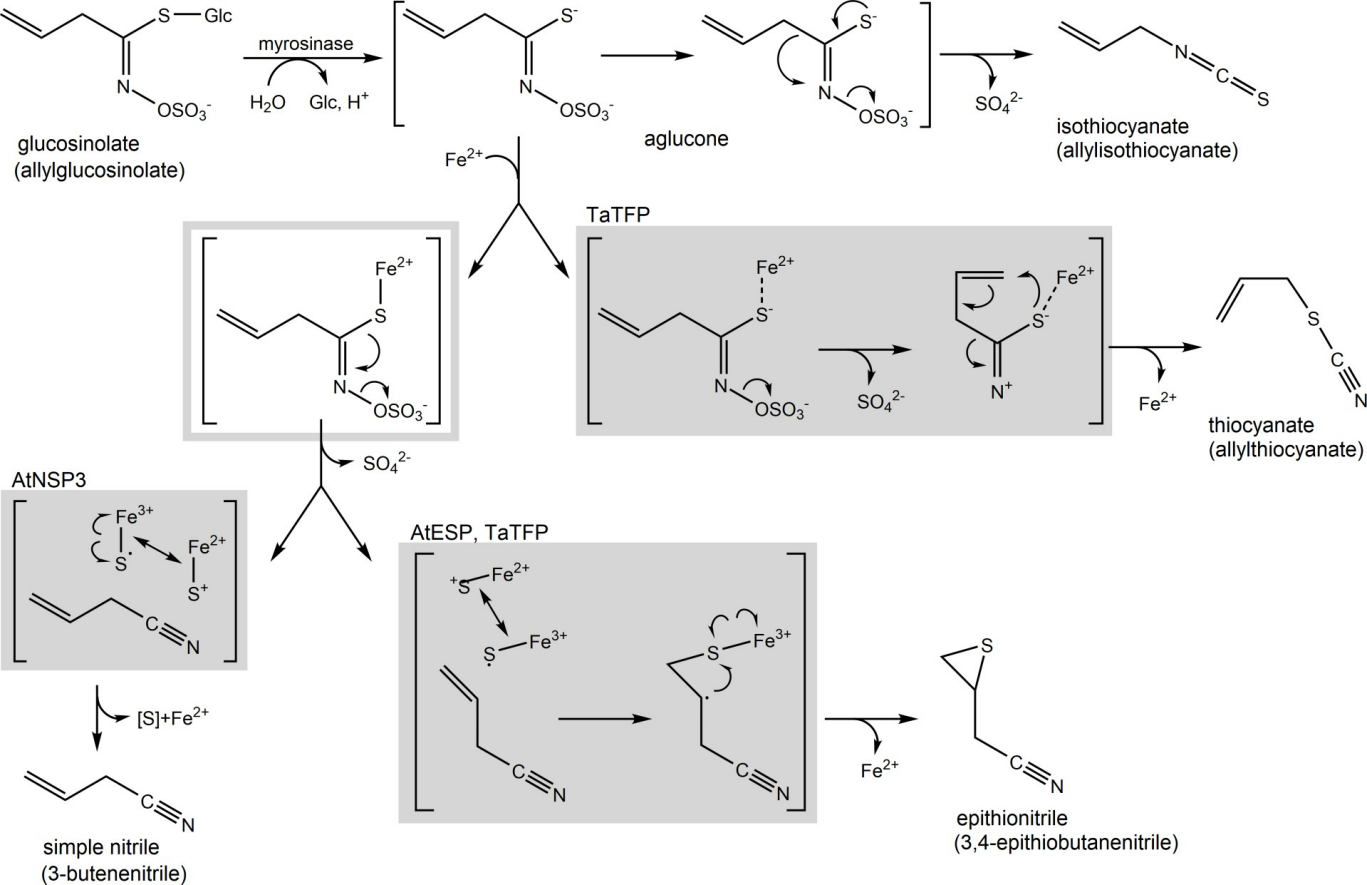
Glucosinolate breakdown pathways. Pathways are illustrated using allylglucosinolate as an example. Myrosinase-catalyzed glucosinolate hydrolysis yields an unstable aglucone that undergoes a spontaneous Lossen-like rearrangement to the isothiocyanate. In the presence of specifier proteins, alternative breakdown products (simple nitrile, epithionitrile, thiocyanate) are formed. Boxes indicate hypothetic mechanisms supposively mediated by specifier proteins. Specifier proteins analyzed in the present study are assigned to the boxes. If the aglucone lacks the structural requirements for epithionitrile and/or thiocyanate formation, all types of specifier proteins promote simple nitrile formation (not shown). For more detailed explanations, see text. Boxed reaction schemes are redrawn from [20] and [21] with modifications.

Glucosinolates are a group of well-studied amino acid-derived thioglucosides found in plants of the order Brassicales including agriculturally important crops of the Brassicaceae [1–3]. As components of an activated plant defense system, the 'mustard oil bomb', glucosinolates are accumulated separately from their hydrolyzing enzymes, thioglucosidase glucohydrolases commonly referred to as myrosinases, within the intact plant [3, 4]. Tissue disruption, e.g. by herbivore attack, destroys compartmentation and thus initiates myrosinase-catalyzed hydrolysis of glucosinolates to deterrent and toxic chemicals which fight off the attacker [1]. Among the various products of glucosinolate breakdown, the isothiocyanates (mustard oils; Fig 1) have most frequently been demonstrated to have direct negative effects on plant enemies such as microbes, nematodes, and insects [5–7] and have also attracted a lot of interest as flavor and health-promoting compounds formed upon ingestion of vegetables and spices such as broccoli, horseradish and nasturtium by humans [8–10]. Other types of breakdown products include nitriles, epithionitriles and organic thiocyanates (Fig 1) whose formation is often restricted to certain plant organs or developmental stages and regulated by environmental conditions [11–13]. Their biological roles are less well understood than those of isothiocyanates and may include their involvement in direct as well as indirect defense responses [4]. Besides breakdown upon tissue disruption, glucosinolates can also be hydrolyzed in undamaged tissue in response to pathogen attack, but also in response to sulfur deficiency or during development, indicating additional roles beyond plant defense and the existence of specifically regulated breakdown pathways [14–19].

Isothiocyanates are produced upon spontaneous rearrangement of the unstable aglucone following glucosinolate hydrolysis, irrespective of the glucosinolate side chain (Fig 1). In contrast, the formation of simple nitriles, epithionitriles and organic thiocyanates depends on the side chain structure and appears to require additional proteins under physiological conditions [22] (Fig 1). The first description of such a protein, the epithiospecifier protein (ESP) from *Crambe abyssinica* (Brassicaceae), dates back to 1973 when ESP activity was separated from myrosinase activity of *C*. *abyssinica* seed meal [23]. It was shown to require Fe^2+^ for activity and to be necessary in addition to myrosinase activity to produce the epithionitrile derived from allylglucosinolate (3,4-epithiobutane nitrile) [23]. This means that specifier proteins have no hydrolytic activity on glucosinolates, but affect the outcome of myrosinase-catalyzed glucosinolate hydrolysis. More recent research has identified genes encoding three different types of plant specifier proteins, i.e. ESPs, thiocyanate-forming proteins (TFPs), and nitrile-specifier proteins (NSPs), which are classified based on their product profiles [13, 24–29] (Fig 1). TFPs promote the formation of organic thiocyanates from glucosinolates with an allyl-, benzyl- or 4-methylthiobutyl side chain, epithionitrile formation from alkenylglucosinolates and simple nitrile formation from all structural types of glucosinolates. Known TFPs differ in their substrate/product specificity with respect to thiocyanate formation from allyl- and benzylglucosinolate, i.e. TFP from *Thlaspi arvense* (Brassicaceae; TaTFP) produces organic thiocyanate only upon hydrolysis of allylglucosinolate while TFP from *Lepidium sativum* (Brassicaceae; LsTFP) produces organic thiocyanate only upon hydrolysis of benzylglucosinolate [26, 29]. When compared to TFPs, ESPs lack the ability to promote thiocyanate formation while NSPs lack both thiocyanate and epithionitrile-forming activity.

Based on their substrate/product specificity, specifier proteins are likely enzymes which act on the glucosinolate aglucone (Fig 1). This is, however, difficult to prove because the aglucone is a short-lived intermediate that cannot be isolated for use as substrate in enzyme assays or for crystallization. Moreoever, kinetic parameters cannot be determined for specifier proteins, and activity assays can only be conducted in combination with myrosinase as the hydrolytic enzyme. Previous labeling studies have shown that the sulfur of the epithionitrile's thiirane ring originates from the thioglucosidic sulfur [30] while thiocyanate formation from allylglucosinolate is associated with a rearrangement of the side chain in agreement with an attack of this sulfur at the terminal carbon atom [21] (Fig 1). Increased activity upon supplementation of *in vitro* assays with Fe^2+^ has been reported for all three types of specifier proteins [25, 26, 28, 29, 31, 32]. Experiments with purified recombinant ESP from *Arabidopsis thaliana* (Brassicaceae; AtESP) showed that Fe^2+^ leads to a stronger activation than Fe^3+^ while other metal ions are unlikely to act as cofactors [31]. Consequently, a possible role of specifier proteins could be to support the correct positioning of an iron cofactor relative to the aglucone sulfur, to preserve their required charges and to stabilize reactive conformations of the aglucone side chain [20, 22].

Amino acid sequence analysis identified several repeats of the so-called Kelch motif (indicating a propeller-like structure) in all known specifier proteins and one or two N-terminal jacalin-related lectin (JAL) domains in some, but not all NSPs [24] [13]. Based on previous molecular modeling efforts [33], the recent elucidation of the crystal structures of TaTFP as well as of AtESP and NSP1 from *A*. *thaliana* (AtNSP1) showed that specifier proteins, in fact, adopt a six-bladed β-propeller fold defined by the kelch repeats [34–36]. In addition, AtNSP1 possesses an N-terminal JAL domain with a β-prism fold [36].

When the first molecular models were established, potential iron binding sites had been evaluated in order to define an active site, and an iron ion had been included manually at the site with the highest scores [33]. Mutational analysis demonstrated that each of the proposed iron-binding residues E266, D270, and H274 of TaTFP is essential for TaTFP activity [33, 34]. However, none of the crystal structures contained an iron cofactor despite supplementation of the crystallization buffer with Fe^2+^ in case of TaTFP [34–36]. Therefore, an iron was inserted at the previously proposed site by molecular modeling, and the obtained TaTFP structure was used for further docking studies with glucosinolate aglucones. In support of the proposed active site, this resulted in high quality docking arrangements with an ideal quadratic bipyramidal coordination of the iron by one oxygen of E266 and D270, a nitrogen of H274, the aglucone sulfur atom as well as two water molecules, and sufficient stabilization of the aglucone side chain [34]. Similar docking arrangements were obtained using the crystal structures of AtESP and AtNSP1 [35, 36]. Substitution of the corresponding iron binding residues of AtESP, E260 and D264, by Gln and Asn, respectively, resulted in strongly diminished activity confirming the importance of these amino acid residues [33].

Taken together, iron ions are very likely involved in the mechanism of specifier proteins based on the present knowledge, but the ultimate proof of their presence in specifier proteins is still lacking. The aim of this study was to test the hypothesis that specifier proteins harbor a specifically bound iron with an essential role for activity. We applied two different techniques to assess iron content in purified recombinant specifier proteins of three different types (TFP, NSP, ESP). In addition to wildtype proteins, we also analyzed proteins with amino acid substitutions in the proposed iron-binding site to provide a link to the structural information available from previous studies and from an AtNSP3 homology model described here.

## Methods

### General

Myrosinase was purified from seeds of *Sinapis alba* (Brassicaceae) as described before [29]. Bovine serum albumin (BSA), chicken egg ovalbumin and human holo-transferrin were purchased from Applichem. Ferene S [37] was purchased from Sigma-Aldrich. Protein content was determined with the Pierce BCA Protein Assay kit (Thermo Fisher Scientific) using BSA as a standard.

### Expression constructs

All expression constructs used in this study were based on modified pET52b(+) vector (Novagen) as described in [29] and enabled production of recombinant proteins with an N-terminal Strep-Tag II followed by an HRV 3C cleavage site. Expression constructs for AtESP (At1g54040), TaTFP (Genbank JN244735), TaTFP E266Q, and TaTFP D270N are described in [33], the construct for TaTFP H274G is described in [34]. The expression construct for AtNSP3 (At3g16390) [27] was used as a template to generate AtNSP3 E386Q, AtNSP3 D390N, and AtNSP3 H394A by site directed mutagenesis as described previously [33] using the following primers: 5'-TGATATTTGGAGGTCAGATTGCGATGG (E386Q), 5'-TGAGATTGCGATGAATCCACGAGCTCAC (D390N), 5'-ATGGATCCACGAGCTGCCGTATCCG (H394A). Open reading frames of all constructs used in this study were confirmed by sequencing.

### Expression and purification

Specifier proteins were expressed in *E*. *coli* as described previously [29], but expression cultures were grown in enriched medium (2.4% (w/v) yeast extract, 1.6% (w/v) tryptone, 1% (w/v) casamino acids, 1% (w/v) glycerol and 100 mM MS, pH 7.4) for 40 h in case of AtNSP3 and its mutants. Crude extracts were loaded onto Strep-Tactin Sepharose or Strep-Tactin XT for purification of the recombinant proteins according to the instructions by the manufacturer (IBA, Göttingen, Germany). Deviating from the manufacturer’s directions, no EDTA was added to the elution buffer. Purity of the fractions was analyzed by Tris/SDS-PAGE (S1 Fig). For use in Ferene S assays and ICP measurements, the buffer was changed to 37.5 mM sodium acetate, pH 5, with 6.25% (w/v) sucrose (TaTFP) or 50 mM MES, pH 6.5, (AtESP and AtNSP3) using PD Mini Trap G-25 columns (GE Healthcare).

### Specifier protein activity

Purified protein (in 100 μl elution buffer) was incubated with 2 mM allylglucosinolate or 1 mM benzylglucosinolate, 0.01 mM (NH_4_)_2_Fe(SO_4_)_2_ and 0.005 units myrosinase (added at last) in 50 mM MES buffer, pH 6, in a total volume of 500 μl at 22°C. After 40 min, 50 μl of phenylcyanide (100 ng μl^-1^ in MeOH) were added as internal standard, and glucosinolate breakdown products in dichloromethane extracts of the assay mixture were quantified by GC-FID as described previously [34]. To test the effect of Fe^2+^ vs. Fe^3+^, reactions were conducted as described above using 30 μg purified TaTFP and 0.01 mM Fe^2+^ supplied as (NH_4_)_2_Fe(SO_4_)_2_ or 0.01 mM Fe^3+^ supplied as NH_4_Fe(SO_4_)_2_.

### Ferene S assay

The assay was conducted as described in [38], but without preincubation with iron. Purified recombinant specifier proteins (200 μl of 0.4–2 mg/ml, corresponding to 2–10 nmol monomers) were mixed with 30 μl concentrated HCl and incubated for 10 min at 25°C with rotation on an end-over-end shaker. After addition of 25 μl 80% (w/v) trichloroacetic acid and 10 min incubation on ice, samples were centrifuged at 10000 x g for 10 min. Acetic acid (45% (v/v), 50 μl) was added to 225 μl of the supernatant followed by addition of 450 μl Ferene S reagent (10 mM L-ascorbic acid, 0.75 mM Ferene S in 45% (w/v) sodium acetate) resulting in a final pH of 5.5–5.6, depending on the buffer of the protein solution. After incubation at room temperature for 10 min, 200 μl were transferred to microplates and the absorbance at 595 nm was measured on a Tecan microplate reader (Crailsheim, Germany). As a control, ovalbumin and holo-transferrin were dissolved at 2.5 nmol/200 μl in the same buffer as the protein of interest and treated the same. To generate a calibration line, 1–25 nmol (NH_4_)_2_Fe(SO_4_)_2_ were dissolved in 200 μl of the same buffer as the protein of interest and subjected to the same procedure. The absorbance of the blank (same buffer with no Fe addition) was deduced as background from all other values. To ensure that measurements were conducted in the linear range, different concentrations of TaTFP were subjected to the procedure (Figure A in S2 Fig). Proteins represented in the same graph were analyzed in parallel.

### Inductively Coupled Plasma-Mass Spectrometry (ICP-MS)

Protein samples were processed according to [39] without preincubation with iron. Specifier proteins (200 μl of 0.4–2 mg/ml, corresponding to 2–10 nmol monomers) were mixed with 250 μl HNO_3_ (Rotipuran Supra 69% (w/w), Roth) and left overnight. After addition of 80 μl hydrogen peroxide solution (≥ 30% (w/w) for ultratrace analysis, Sigma) and incubation in an ultrasonic bath at 60°C for 2 h, samples were diluted tenfold with deionized water (≥ 18.2 MΩcm). As a control, ovalbumin and holo-transferrin were dissolved at 2.5 nmol/200 μl in the same buffer as the protein of interest and treated the same. Measurements were conducted using an Agilent ICP-MS 7700x controlled by MassHunter software. ICP multielement standards (Roth or Fluka) were used as operational controls and Rhodium ICP-MS standard (Fluka) as internal standard at 10 μg/l. ^56^Fe content was calculated based on a calibration line obtained with a dilution series (0–20 μg/l Fe) of Iron ICP Standard Solution (1000 mg/l Fe, Roth or Fluka) in deionized water (≥ 18.2 MΩcm) supplemented with 10 μg/l BSA using the instrument's software. The blank value obtained with no Fe addition was deduced as background from all other values. To ensure that measurements were conducted in the linear range, different concentrations of TaTFP were subjected to the procedure (Figure B in S2 Fig). Proteins represented in the same graph were analyzed in parallel.

### Molecular modeling

Generation of AtNSP3 homology models was performed with YASARA Version 17.12.24 (www.yasara.org) [40, 41] using the predefined template structures TaTFP (PDB 5A10, [34]) and AtNSP1 (PDB 5GQT, [36]). For preparation of hybrid structures the JAL domain (amino acids 1–146) and Kelch domain (amino acids 147–467) were modeled separately. Fe^2+^, water and docked allylglucosinolate aglucone of TaTFP template were inherited to incorporate their effects during the propeller domain modeling. The obtained domain models were structurally refined with short molecular dynamic simulations at 298 K and pH 7.4 using Yasara2 force field in a simulation cell with periodic boundary conditions, filled with explicit water and neutralized by NaCl counter ions. Domains were linked, further optimized and energy minimized and finally evaluated regarding their folding behavior and geometry with PROSA II [42] and PROCHECK [43], respectively. The best evaluated AtNSP3 model was transferred into GB implicit water for an improvement of the Fe^2+^ coordination complex with Amber12:EHT force field in MOE 2016.08 (Molecular Operating Environment, Chemical Computing Group Inc., 2016). Docking of the allylglucosinolate aglucone was done in GOLD [44, 45] using the Gold Score fitness function for evaluation. Amino acids inside a radius of 15 Å around the center [20.09, 109.19, 24.98], which was located adjacent to C_ζ_ of R237 guanidino group, formed the active site. For N188, F271, R237, R292 and H394 side chain flexibility regarding the GOLD rotamer library was taken into account. Fe^2+^ was described as octahedral coordinated cofactor. Water molecules involved in the binding site were always present during the docking procedure. Fifty different docking positions were generated. Resulting protein ligand complexes were energy minimized with Amber12:EHT in GB implicit water and their interaction energies regarding AtNSP3 were calculated.

### Statistics

Statistical analysis was conducted with OriginPro 8.0. The assumption of normally distributed errors was tested using the Shapiro-Wilk test. Equal variance was assumed based on the Brown-Forsythe test. ANOVA with post-hoc Tukey's test was used to identify significant differences between treatments if more than two treatments were compared. If the errors were found to be non-normal, the Kruskal-Wallis non-parametric test was applied instead of ANOVA. To test the impact of Fe^2+^ supplementation on total product formation by myrosinase and TaTFP, the Mann-Whitney test was performed as errors were found to be non-normal.

## Results

**Fig 2:**
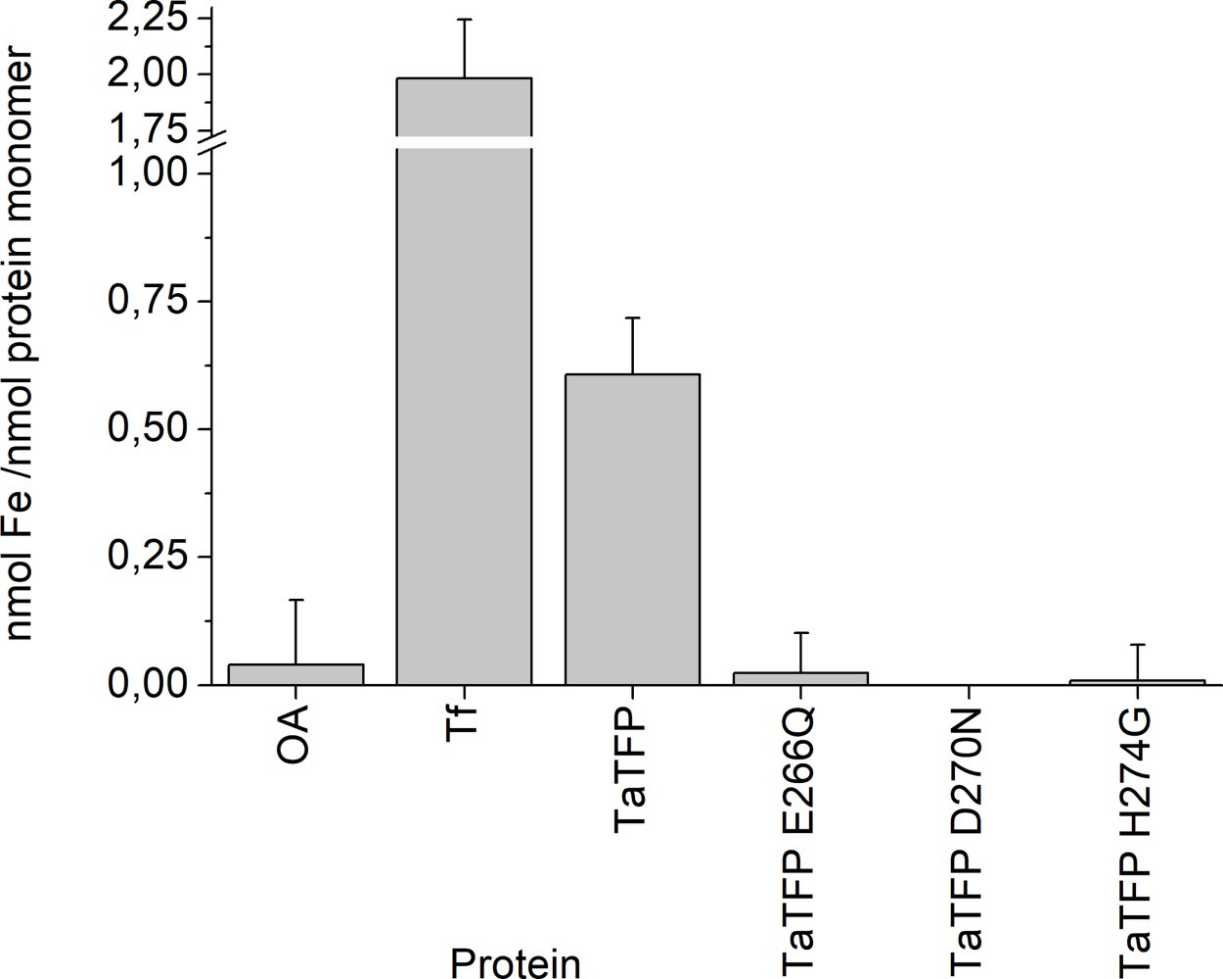
Ferene S quantification of iron in TaTFP wildtype and mutants. Proteins (in 37.5 mM sodium acetate buffer, pH 5, with 6.25% (w/v) sucrose) were denatured and precipitated. Fe^2+^ in the supernatant was quantified colorimetrically using Ferene S reagent and calibration with (NH_4_)_2_Fe(SO_4_)_2_. OA, ovalbumin; Tf, holo-transferrin. Shown are means ± SD (N = 6 independent expression experiments).

To investigate if TaTFP harbors a centrally bound iron atom, we followed a previously described protocol [38] to use Ferene S, an iron-specific chelator [37], for colorimetric detection of iron released upon protein denaturation. As Ferene S forms stable, water-soluble complexes with Fe^2+^, reducing conditions were applied to keep the released iron in the Fe^2+^ state. We found purified recombinant TaTFP to contain, on average, 0.6 nmol iron per nmol protein monomer without preincubation with iron (Fig 2). To validate the experimental setup, we also quantified iron in holo-transferrin with two specifically bound Fe^3+^ per molecule [46] and in ovalbumin as negative control. On average, we detected 2.0 nmol iron per nmol holo-transferrin and 0.04 nmol iron per nmol ovalbumin (Fig 2), confirming our expectation. As TaTFP activity increases upon supplementation of enzyme assays with Fe^2+^ [29] (S3 Fig), one possible explanation would be that TaTFP binds iron, but partially loses it upon protein purification. The only partial loss of iron would be in accordance with previous experiments in which TaTFP was still active in enzyme assays supplemented with 0.1 mM Fe^2+^ and 10 mM EDTA [29]. When we preincubated TaTFP with 0.1 or 0.01 mM Fe^2+^ to reach saturation, we obtained similar values as without preincubation, likely due to partial removal of bound iron by the gel filtration step that we had to include to remove unbound iron. As an alternative explanation, TaTFP may exist in an iron binding-competent and an iron binding-incompetent form. If a fraction of the protein remains in the iron binding-incompetent form, saturation with Fe^2+^ may only be achievable for the iron binding-competent fraction.

To test if the previously proposed iron-binding residues of TaTFP [34] affect the capacity of TaTFP to bind iron, we also subjected mutant proteins to the Ferene S assay in which E266, D270, or H274 were substituted by the corresponding amide or Gly, respectively. On average, we detected levels ≤ 0.02 nmol iron per nmol protein for the mutants, similar to the background levels obtained with ovalbumin (Fig 2). As the investigated mutants are correctly folded, but enzymatically inactive [34], we concluded that the proposed iron-binding residues E266, D270, and H274 of TaTFP are in fact essential for the acquisition of iron by TaTFP and that bound iron is required for TaTFP activity.

**Fig 3:**
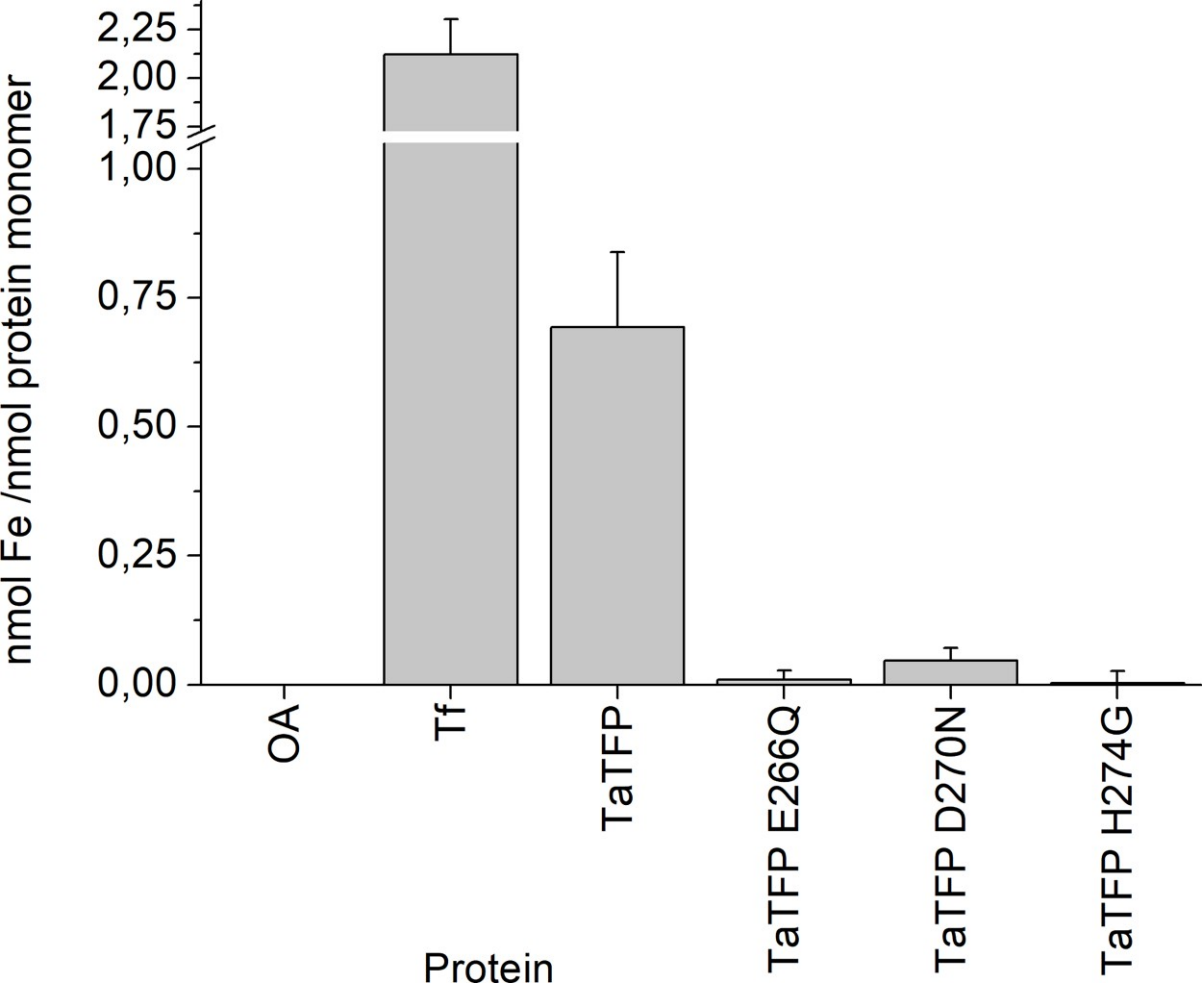
ICP-MS quantification of iron in TaTFP wildtype and mutants. Proteins (in 37.5 mM sodium acetate buffer, pH 5, with 6.25% (w/v) sucrose) were denatured and precipitated. The supernatant was subjected to ICP-MS. Iron was quantified based on calibration with Iron ICP Standard. OA, ovalbumin; Tf, holo-transferrin. Shown are means ± SD (N = 3 independent expression experiments).

To further substantiate our findings, we applied ICP-MS to specifically detect and quantify iron in the supernatant obtained after denaturation and precipitation of TaTFP and the derived mutants. Similar to the results obtained with Ferene S, we detected, on average, 0.7 nmol iron per nmol TaTFP monomer but ≤ 0.05 nmol iron per nmol mutant protein monomer (TaTFP E266Q, TaTFP H274G, TaTFP D270N) (Fig 3). Values obtained for the controls, holo-transferrin and ovalbumin, were in the expected range (Fig 3). Thus, the ICP-MS measurements demonstrated that iron is bound to a fraction of roughly two thirds of the TaTFP monomers after heterologous expression and purification. Furthermore, the ICP-MS measurements confirmed that iron binding of TaTFP depends on E266, D270, and H274.

**Fig 4:**
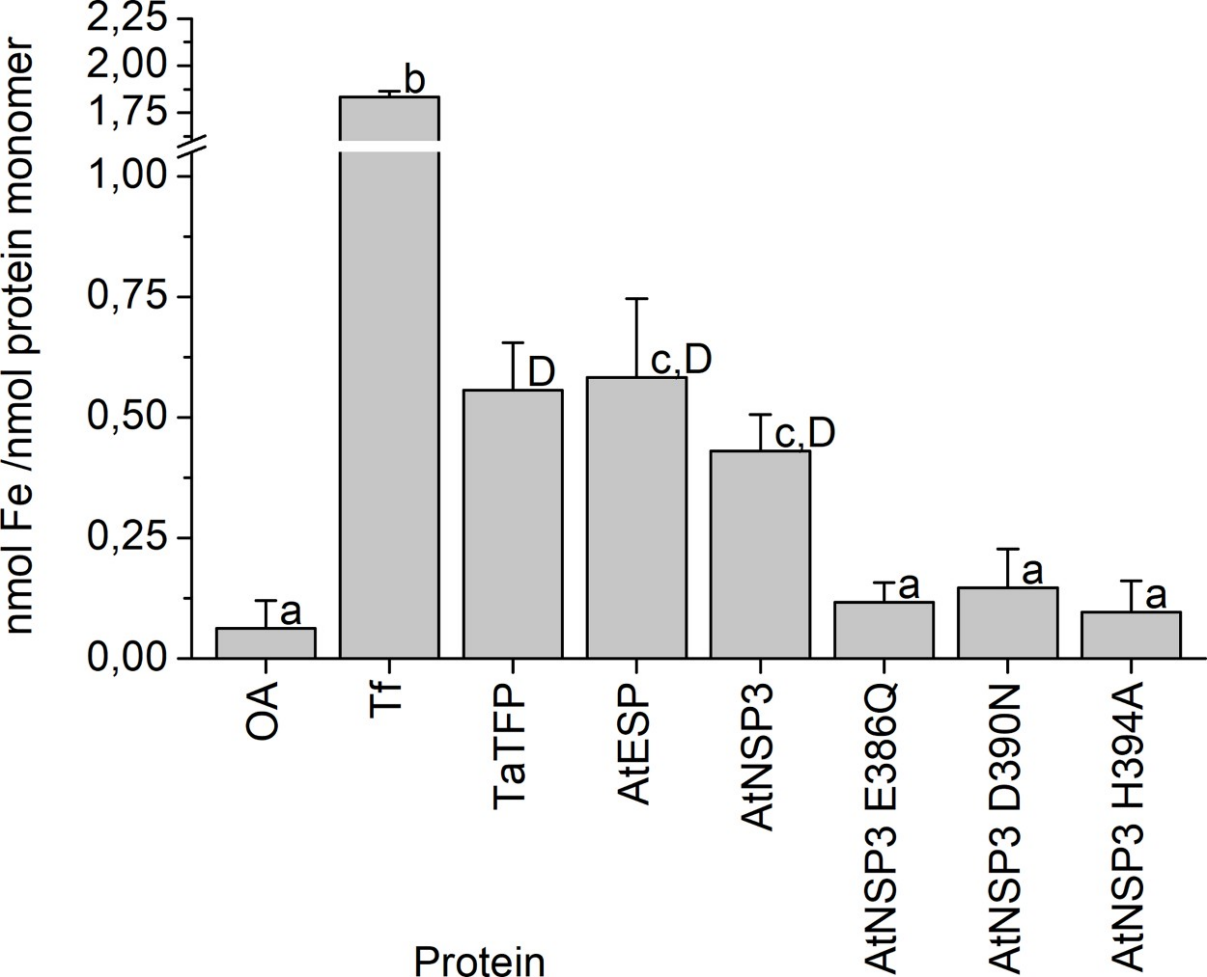
Ferene S quantification of iron in AtESP and AtNSP3 wildtype and mutants. Proteins (in 50 mM MES, pH 6.5, TaTFP in 6.25% (w/v) sucrose in 37.5 mM sodium acetate buffer, pH 5) were denatured and precipitated. Fe^2+^ in the supernatant was quantified colorimetrically using Ferene S reagent and calibration with (NH_4_)_2_Fe(SO_4_)_2_. OA, ovalbumin; Tf, holo-transferrin. Shown are means ± SD (N = 3 independent expression experiments). Different letters above bars indicate a significant difference (p<0.05; lower case letters: ANOVA with Tukey's test (when errors were normally distributed); upper case letter: Kruskal-Wallis non-parametric test (non-normal errors)).

To investigate if the various types of specifier proteins differ in their capacity to bind iron, we compared the iron content of TaTFP, AtESP and AtNSP3 after heterologous expression and purification using the Ferene S assay. We detected iron in all three proteins at levels between around 0.4 and 0.6 nmol per nmol monomer (Fig 4). Although iron content in AtNSP3 (0.4 nmol iron per nmol monomer) was, on average, slightly lower than that of TaTFP and AtESP, this difference was not significant. Thus, these experiments proved the presence of iron in AtESP and AtNSP3, but did not provide indication for different iron binding affinities of TaTFP, AtESP, and AtNSP3.

**Fig 5:**
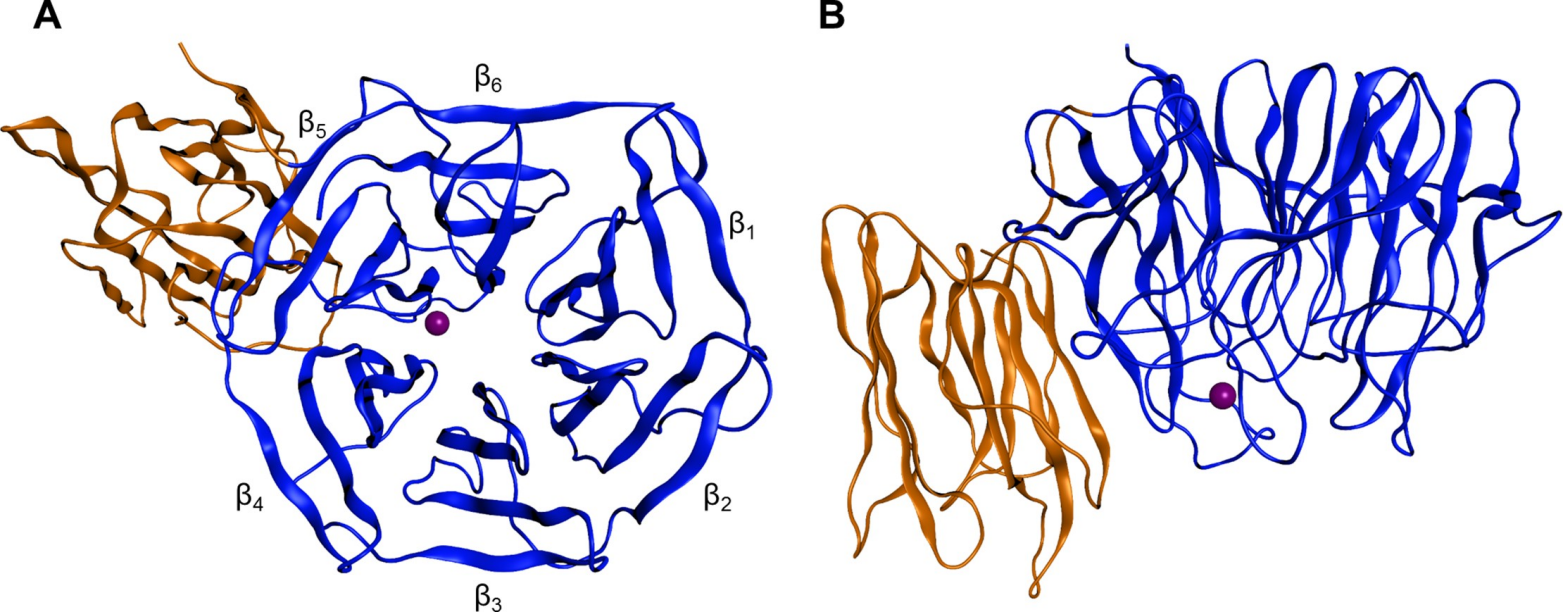
Structure of the final AtNSP3 homology model. The β-propeller and JAL domains are shown in blue and orange, respectively. Fe^2+^ is represented by a purple sphere. (A) Top view. β-Sheets are labeled according to [34]. (B) Side view.

To study iron binding of AtNSP3 in more detail, we generated a molecular model of AtNSP3 using the previously resolved crystal structures of TaTFP [34] and AtNSP1 [36] as templates. Kelch and JAL domains were modeled separately in YASARA. Because of the high resolution of the TaTFP structure (1.42 Å) as compared to the AtNSP1 structure (3.02 Å) and the available docking studies with TaTFP [34], the Kelch domain of AtNSP3 was modeled using the TaTFP monomer despite an amino acid sequence identity of only 54% (S4 Fig). Molecular modeling of the JAL domain of AtNSP3 was conducted based on the structure of AtNSP1. Due to a high quality of the AtNSP1 structure in this domain according to PROSA II and PROCHECK evaluation and 88% amino acid sequence identity to the JAL domain of AtNSP3 (S4 Fig), we were able to generate a high quality model without extensive refinements. We created a complete AtNSP3 hybrid model by linking the optimized single domain models followed by final geometry optimization and energy minimization in MOE 2016.08 with Amber12:EHT (Fig 5). The orientation of AtNSP3 JAL and Kelch domains was determined by amino acid sequence and structural alignments with AtNSP1. A PROSA II analysis returned an overall combined energy z-score value of -9.91 indicating a native-like fold of the AtNSP3 model. The evaluation with PROCHECK showed that 99.2% of all dihedrals grouped into most favored and additionally allowed regions of the Ramachandran plot. Only one amino acid of the JAL domain was identified as an outlier. Further evaluation scores describing, among others, main chain bond lengths, peptide bond planarity or side chain dihedrals were within ideal value ranges (S1 Table) and were also in agreement with a high quality and suitability of our AtNSP3 model for further analysis. The octahedral binding of Fe^2+^ in TaTFP active site resulted in a conformational change of D270, W309, and the 5L2 loop [34]. As the β-propeller of AtNSP3 was modeled based on the structure of TaTFP with bound Fe^2+^ and allylglucosinolate aglucone, it likely represents AtNSP3 after a similar conformational change.

**Fig 6:**
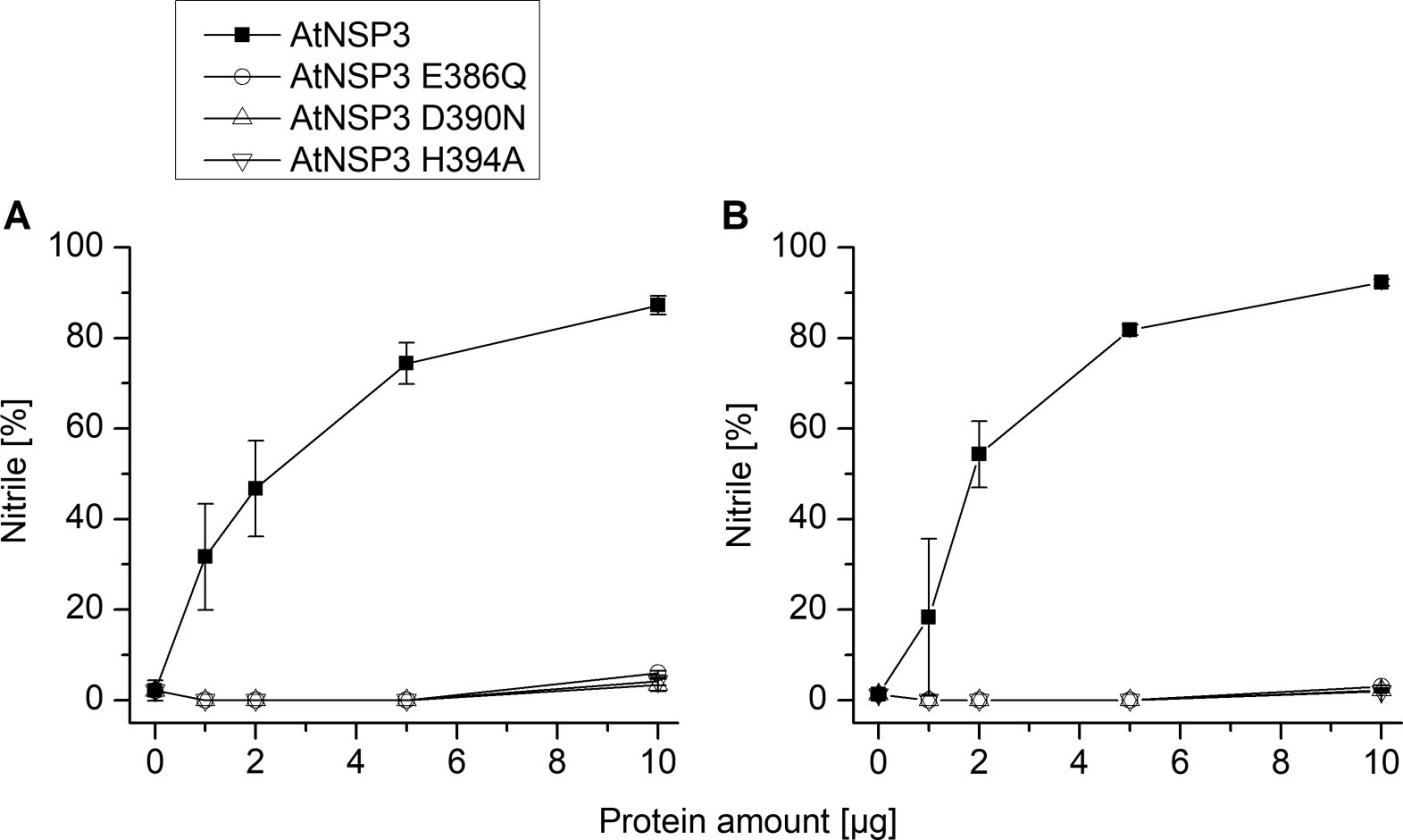
Activity of AtNSP3 mutants with substitutions of proposed iron-binding residues. Purified proteins were incubated with allylglucosinolate (A) or benzylglucosinolate (B) and myrosinase in 50 mM MES buffer, pH 6.0, supplemented with 0.01 mM Fe^2+^ for 40 min. Activity is expressed as the proportion of simple nitrile formed relative to the total amount (nmol) of detected breakdown products. Shown are means ± SD of N = 3 independent expression experiments.

**Fig 7:**
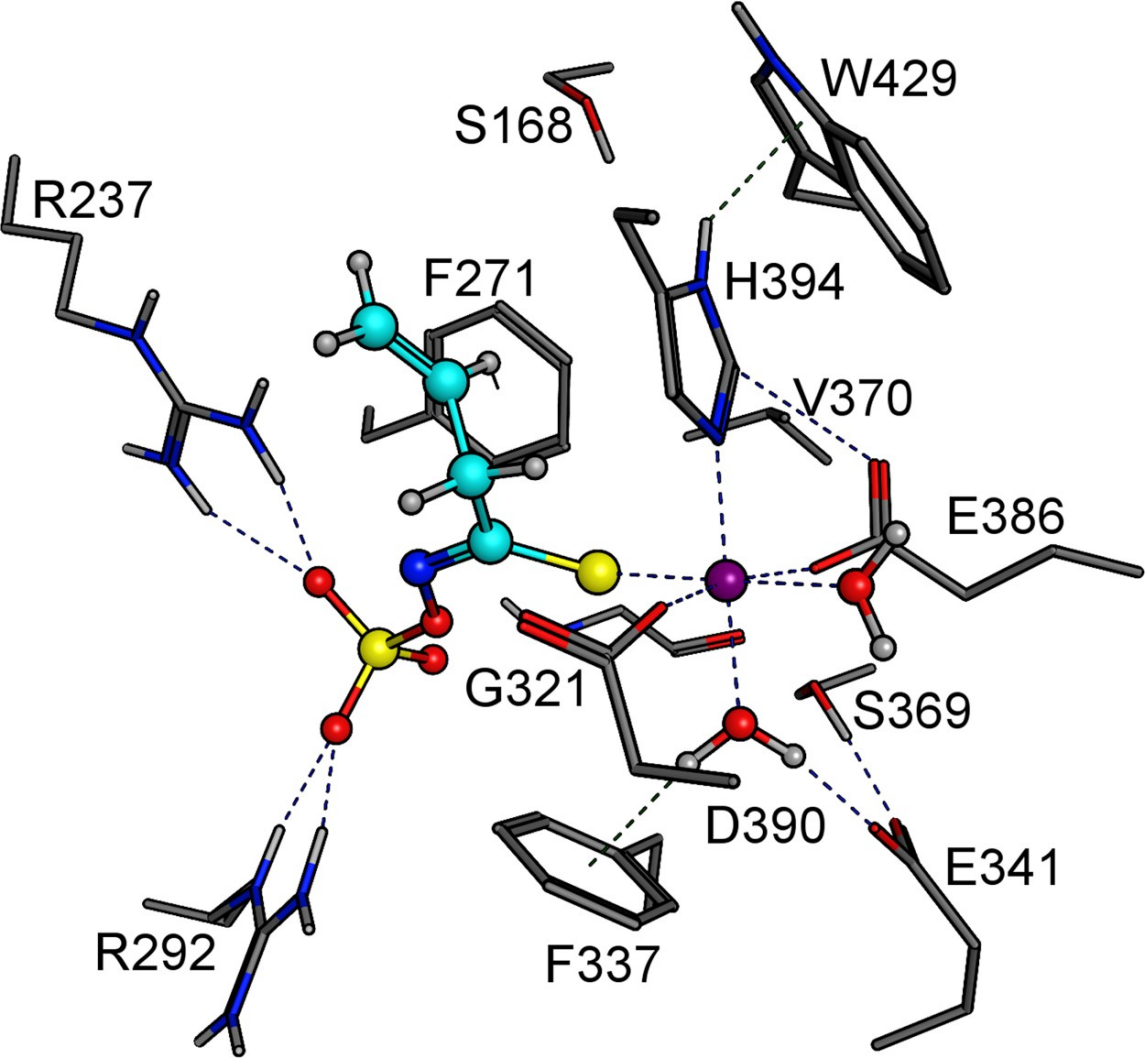
Docking arrangement of AtNSP3 with Fe^2+^ and allylglucosinolate aglucone. Active site residues are shown as sticks with gray C-skeleton. The aglucone is shown with the C-skeleton in cyan. The color code for heteroatoms is as follows: yellow, sulfur; blue, nitrogen; red, oxygen. Fe^2+^ is represented by a purple sphere.

The iron-binding triad of TaTFP (E266, D270, H274) [33, 34] corresponds to E386, D390, and H394 in AtNSP3 (S4 Fig). To test the critical role of these residues experimentally, we determined iron content of mutant proteins in which E386, D390, and H394 are substituted by the corresponding amides or Ala, respectively. Iron content of these mutants was at background levels (Fig 4). Furthermore, the mutant proteins lacked specifier protein activity (Fig 6). This was in agreement with an essential role and conserved binding of the iron cofactor in AtNSP3. To further study the active site of AtNSP3, its octahedral coordination geometry was inherited from the TaTFP template with two introduced water molecules and one unoccupied position for aglucone binding and was optimized during the modeling procedure. The allylglucosinolate aglucone was integrated at the unoccupied position of the trigonal-bipyramidal structure using a protein-ligand docking process with GOLD. Similar to AtNSP1 [36], the strand-connecting loops 3L2 and 4L2 of AtNSP3 are shorter than those of TaTFP and AtESP resulting in a more open binding site. This allowed for a variety of alternative protein-ligand docking arrangements compared to TaTFP and AtESP [33, 34]. Fifty different poses were generated, evaluated based on Gold Score, and subsequently optimized. The obtained protein ligand complexes were ordered regarding their interaction energies. Among the top ten poses, three revealed a reasonable aglucone stabilization with a direct interaction between the iron cofactor and the aglucone (S2 Table). This included the coordination between iron and aglucone thiolate, a sulfate group recognition by at least one Arg residue and a sufficient stabilization of the non-polar aglucone side chain by adjacent amino acids, e.g. F271 and H394 [33] (S2 Table). The most promising complex was again geometry optimized and energy minimized (Fig 7). The final model of AtNSP3 supported the iron coordination by the conserved iron-binding triad (E386, D390, H394) and aglucone binding to the proposed active site by interaction of the aglucone thiolate with the iron cofactor and interaction of the aglucone sulfate with R237 and R292 (Fig 7). The higher accessibility of the active site of AtNSP3 due to shorter 3L2 and 4L2 loops (as compared to TaTFP and AtESP) resulted in less restricted conformations. This may prevent epithionitrile and thiocyanate formation by AtNSP3 which require sufficient stabilization of a specific aglucone conformation (Fig 1). A comparison with the available structure of AtNSP1 shows conservation of most active site residues among TaTFP, AtNSP3 and AtNSP1 (S5 Fig). A high similarity between the active sites of TaTFP, AtNSP3 and AtNSP1 with docked allylglucosinolate aglucone is also supported by the calculated C_α_-RMSD values for the active sites of 0.96 Å (AtNSP3 vs. AtNSP1) and 1.11 Å (AtNSP3 vs. TaTFP).

**Fig 8:**
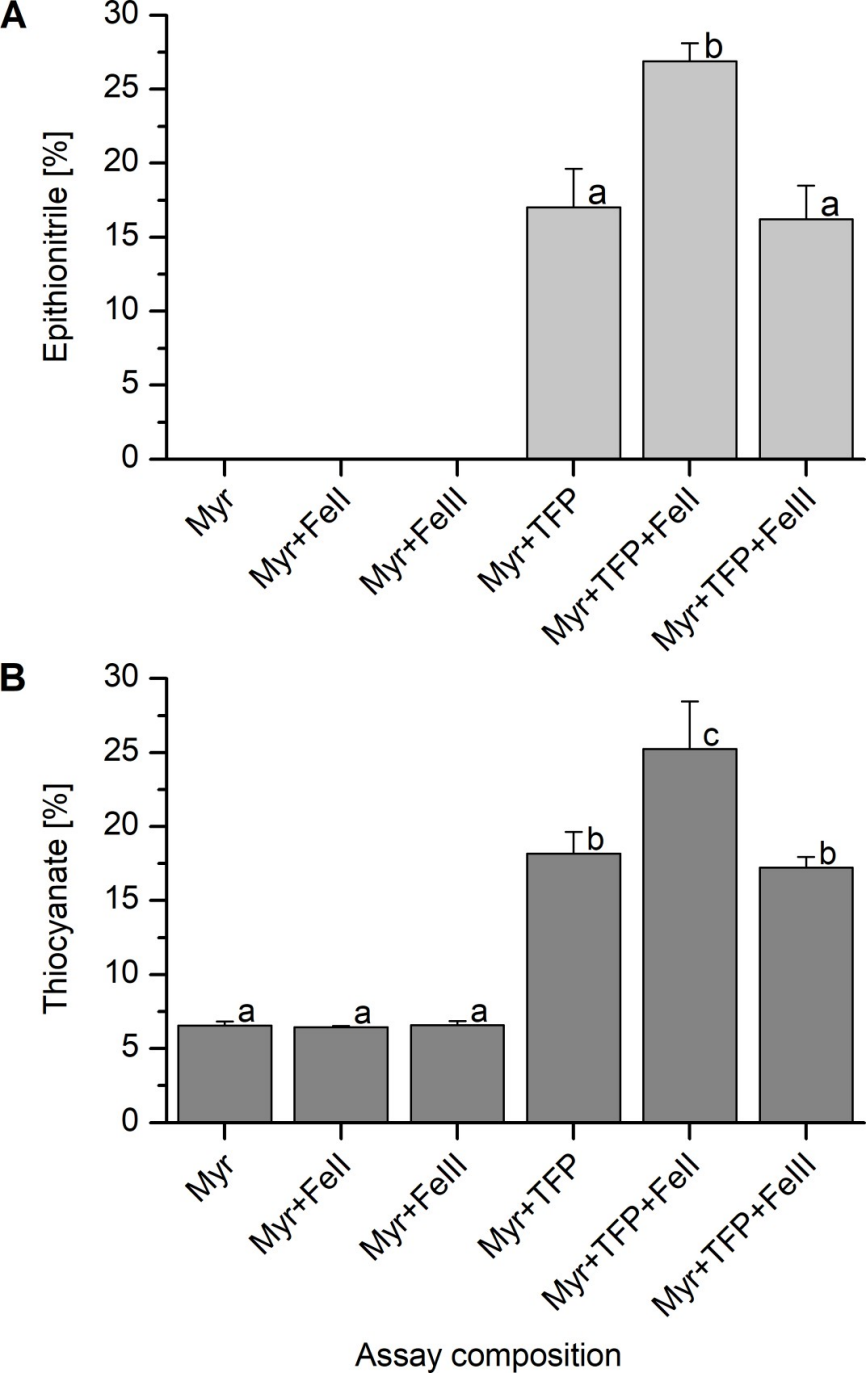
Impact of Fe^2+^ and Fe^3+^ on specifier protein activity of TaTFP. Purified TaTFP was incubated with allylglucosinolate and myrosinase (Myr) in 50 mM MES buffer, pH 6.0, with or without 0.01 mM Fe^2+^ (FeII, (NH_4_)_2_Fe(SO_4_)_2_) or 0.01 mM Fe^3+^ (FeIII, NH_4_Fe(SO_4_)_2_) for 40 min. Activity is expressed as the proportion of individual products ((A) epithionitrile, (B) organic thiocyanate) formed relative to the total amount (nmol) of detected breakdown products. Shown are means ± SD of N = 5 independent expression experiments. Different letters above bars indicate a significant difference (p<0.05; ANOVA with Tukey's test).

The experiments described above did not allow us to draw conclusions on the oxidation state of the bound iron. We therefore compared the effect of added Fe^2+^ and Fe^3+^ on specifier protein activity of TaTFP. In previous studies, concentrations of 0.1 mM Fe^2+^ or above affected the product spectrum of myrosinase (without specifier protein addition) [26, 29]. Therefore, we supplemented the reaction mixtures with 0.01 mM Fe^2+^ or Fe^3+^. Although TaTFP was active without added iron, supplementation with 0.01 mM Fe^2+^ led to a strong and significant increase of the proportion of epithionitrile and thiocyanate formed upon myrosinase-catalyzed hydrolysis of allylglucosinolate in the presence of TaTFP confirming previous results [29] (Fig 8). In contrast, supplementation with 0.01 mM Fe^3+^ did not affect product formation. Product proportions did not change when 0.01 mM Fe^2+^ or 0.01 mM Fe^3+^ was added to control reactions lacking TaTFP (Fig 8), and 0.01 mM Fe^2+^ and 0.01 mM Fe^3+^ had no significant influence on the total amount of hydrolysis products (i.e. myrosinase activity) in the absence or presence of TaTFP (S3 Fig). Hence, Fe^2+^ addition affected TaTFP activity, likely due to assembly of a higher proportion of holo-enzyme, supporting the hypothesis that TaTFP harbors Fe^2+^ as cofactor.

## Discussion

A critical role of an Fe^2+^ cofactor in the reaction mechanisms of simple nitrile, epithionitrile, and thiocyanate formation has long been hypothesized based on effects of iron supplementation. First, it has long been known that addition of Fe^2+^ (but not Fe^3+^) at concentrations ≥ 0.02 mM leads to simple nitrile formation in reactions mixtures containing glucosinolate and myrosinase, but no specifier protein (e.g. [27, 28, 31, 32, 47]. Secondly, specifier protein activities generally increase upon Fe^2+^ supplementation [23, 25, 26, 28, 29, 31, 32]. Thus, iron has repeatedly been found to have a strong impact on product formation upon glucosinolate hydrolysis, but its binding to specifier proteins has not been proven. Our data fill a conceptual gap in the present knowledge on specifier proteins by providing evidence that TaTFP, AtESP and AtNSP3 harbor an iron cofactor, most likely Fe^2+^. Using TaTFP and AtNSP3 we further demonstrate that iron binding depends on the previously proposed, conserved amino acid triad EXXXDXXXH in the central pore of the β-propeller structure. This strongly suggests that iron binding is due to direct interaction of the Fe^2+^ with each of the amino acids E266, D270, and H274 in case of TaTFP and E386, D390, and H394 in case of AtNSP3, which identifies specifier proteins as non-heme iron proteins.

Epithionitrile- and thiocyanate-forming activities of AtESP, TaTFP and TFP from *Lepidium sativum* (Brassicaceae; LsTFP) were reduced or abolished, respectively, upon addition of EDTA, but restored when a surplus of Fe^2+^ was added [26, 29, 31]. In contrast, simple nitrile formation by AtNSP1 was not affected by addition of EDTA [27]. Thus, there is a considerable level of variation with respect to the degree of requirement for Fe^2+^ supplementation and the effects of a chelating agent indicating different Fe^2+^ binding affinities. We, therefore, expected that the various specifier proteins retain different levels of iron during the purification procedure. Our experimental results did not confirm this expectation. We found that the three proteins under investigation, TaTFP, AtESP and AtNSP3, contain about equal levels of iron after purification. It is possible that specifier proteins exist in different conformations that differ in their accessibility to iron binding. Binding and release of Fe^2+^ or Fe^3+^ could also play a role in their reaction mechanisms which, however, still await their elucidation [22, 33, 34] (Fig 1). Experiments to test if Fe^2+^ saturation can be reached when the purified proteins are incubated with Fe^2+^ are not feasible as the excess iron has to be removed before Fe^2+^ measurements with the risk of leakage from the protein. As an alternative, we attempted to use Ferene S as an in-gel detection reagent which does not require prior removal of excess iron [38]. This allowed us to confirm presence of iron in TaTFP upon native PAGE, but quantification was not possible (data not shown). Moreover, other wildtype and mutant proteins precipitated before electrophoresis or did not migrate under the buffer/pH conditions that we had to apply. Future studies should try to determine Fe^2+^ binding constants by alternative techniques such as isothermal titration calorimetry for a quantitative comparison of the Fe^2+^ binding properties of different specifier proteins. This could also be interesting with respect to the evolutionary ancestors of specifier proteins represented by the *At3g07720* locus in *A*. *thaliana* [13]. *At3g07720* has been proposed to play a role for plant iron homeostasis based on its strong induction upon iron deficiency [48–50]. The conserved iron-binding triad of specifier proteins (EXXXDXXXH) is present in modified form in the protein encoded by *At3g07720* (EXXXSXXXH) [33]. This is likely to reduce its Fe^2+^ affinity compared to specifier proteins. Our attempts to determine iron content in At3g07720 have failed so far due to difficulties in obtaining sufficient protein amounts. It is, however, conceivable, that a stronger Fe^2+^ binding might have been a prerequisite for neofunctionalization, i.e. evolution of the ability to form non-isothiocyanate products upon glucosinolate hydrolysis, the function of specifier proteins.

NSPs are the evolutionary oldest specifier proteins [13]. The presence of five NSP genes in the genome of *A*. *thaliana* might seem surprising given the fact that simple nitriles are also formed in the absence of specifier proteins at pH < 5 or Fe^2+^ concentrations ≥ 0.02 mM *in vitro* [27, 28, 31, 32, 51]. However, such conditions are unlikely to affect product formation under physiological conditions *in planta* or upon tissue damage. Thus, plants probably gained selective advantage from possessing NSPs, and their differential expression allowed them to control product profiles tightly in response to environmental challenges [27]. Our data indicate that the composition of the iron-binding site of specifier proteins ensures tight enough Fe^2+^ binding to maintain a high proportion of holo-enzyme independently of the concentration of free iron in the storage compartment (likely the cytosol, [52]) or in crushed tissue. In agreement with the crystal structure of AtNSP1 [36], our homology model of AtNSP3 indicates that the NSP active site is more open than that of TaTFP and AtESP and therefore unable to support epithionitrile or organic thiocyanate formation. Future studies should address this in more detail and investigate the structural bases of substrate/product specificities of ESPs vs. TFPs. *In silico* investigations on a semiempirical or *ab initio* level should be applied to elucidate the molecular mechanisms of product formation and the role of the iron cofactor. Such investigations would greatly benefit from knowledge about the spin state of the iron cofactor and electron transitions. Insights into the mechanisms of product formation by specifier proteins will not only shed light on the evolution of this function, which has developed specifically in the Brassicales, but may also reveal possibilities for applications of these interesting non-heme iron proteins or derived variants, e.g. as biocatalysts in chemical production processes.

## Supporting information

S1 Fig SDS-PAGE analysis of purified recombinant proteins used in this study. Coomassie-stained 10.5% SDS-polyacrylamide gel with 2 μg protein per lane. 1, AtNSP3; 2, AtNSP3 E386Q; 3, AtNSP3 D390N; 4, AtNSP3 H394 A; 5, AtESP; 6, TaTFP; 7, TaTFP E266Q; 8, TaTFP D270N; 9, TaTFP H274G.
(PDF)

S2 Fig Linearity of iron determinations of TaTFP. Different amounts of TaTFP (in 37.5 mM sodium acetate buffer, pH 5, with 6.25% (w/v) sucrose) were denatured and precipitated. (A) Fe^2+^ in the supernatant was quantified colorimetrically using Ferene S reagent and calibration with (NH_4_)_2_Fe(SO_4_)_2_. (B) Iron in the supernatant was quantified by ICP-MS based on calibration with Iron ICP Standard. Each panel shows the results of three independent expression experiments.
(PDF)

S3 Fig Impact of Fe^2+^ and Fe^3+^ on product formation by myrosinase and TaTFP. Purified TaTFP was incubated with allylglucosinolate and myrosinase in 50 mM MES buffer, pH 6.0, with or without 0.01 mM Fe^2+^ (FeII, (NH_4_)_2_Fe(SO_4_)_2_) or 0.01 mM Fe^3+^ (FeIII, NH_4_Fe(SO_4_)_2_) for 40 min. The amount of breakdown products is given in nmol per reaction. Shown are means ± SD of N = 5 independent expression experiments. The p value is given for a pairwise comparison of total amounts of product (Mann-Whitney test as errors were found to be non-normal).
(PDF)

S4 Fig Amino acid sequence identity among TaTFP, AtNSP3, and AtNSP1. Positions shown as active site residues of AtNSP3 in S5 Fig are highlighted with blue background if identical among all three sequences and with cyan background if identical in only two sequences. The proposed Fe^2+^-binding triad is marked with asterisks above the alignment. Among the other positions, identity of the three sequences is indicated by black background, amino acid similarity by gray background. Amino acid sequences were aligned using ClustalW (https://embnet.vital-it.ch/software/ClustalW.html). Shading was introduced by Boxshade (https://embnet.vital-it.ch/software/BOX_form.html) and color-edited manually.
(PDF)

S5 Fig Active site of AtNSP3 in comparison with those of TaTFP and AtNSP1. (A) Model of the AtNSP3 active site with docked Fe^2+^ and allylglucosinolate aglucone. (B) AtNSP1 active site as represented in the crystal structure (PDB 5GQT, [36]). (C) TaTFP active site with docked Fe^2+^ and allylglucosinolate aglucone [34] derived from the crystal structure (PDB 5A10). An alignment of the three amino acid sequences is provided in S4 Fig.
(PDF)

S1 Table Model quality of AtNSP3 evaluated with PROCHECK. Main chain parameters comprises the overall Ramachandran plot quality, peptide bond planarity, hydrogen bond energy, bad non-bonded interactions, and G-factor. Side chain parameters comprises chi1 and chi2 dihedrals.
(PDF)

S2 Table Evaluation of GOLD docking poses obtained with AtNSP3, Fe^2+^ and allylglucosinolate aglucone. The ten poses with lowest AtNSP3-aglucone interaction energies are included. Fulfillment of the three essential docking criteria is indicated.
(PDF)
