# Supplementary material for: Iron is a centrally bound cofactor of specifier proteins involved in glucosinolate breakdown
Source: PLoS One. 2018 Nov 5;13(11):e0205755. doi: 10.1371/journal.pone.0205755 (PMC6218027; doi:10.1371/journal.pone.0205755)
Supplement: S1 Fig — Coomassie-stained 10.5% SDS-polyacrylamide gel with 2 μg protein per lane. 1, AtNSP3; 2, AtNSP3 E386Q; 3, AtNSP3 D390N; 4, AtNSP3 H394 A; 5, AtESP; 6, TaTFP; 7, TaTFP E266Q; 8, TaTFP D270N; 9, TaTFP H274G. (PDF) [file pone.0205755.s001.pdf]

Iron is a centrally bound cofactor of specifier proteins involved in glucosinolate breakdown

Anita Backenköhler, Daniela Eisenschmidt, Nicola Schneegans, Matthias Strieker, Wolfgang Brandt, and Ute Wittstock

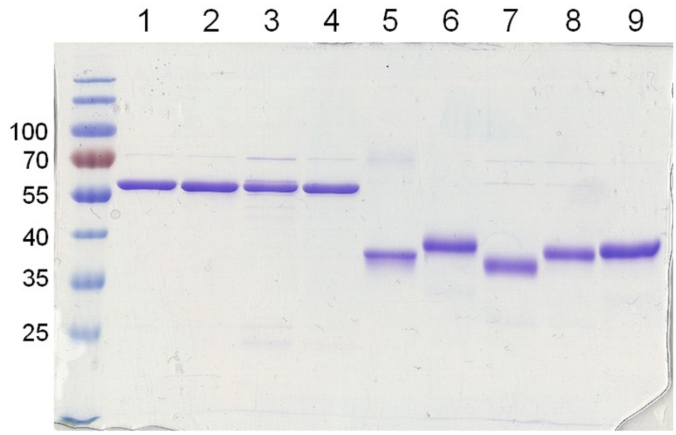

S1 Fig. SDS-PAGE analysis of purified recombinant proteins used in this study.

Coomassie-stained 10.5 % SDS-polyacrylamide gels with 2 µg protein per lane. 1, AtNSP3; 2, AtNSP3 E386Q; 3, AtNSP3 D390N; 4, AtNSP3 H394A; 5, AtESP; 6, TaTFP; 7, TaTFP E266Q; 8, TaTFP D270N; 9, TaTFP H274G.
