# Supplementary material for: Iron is a centrally bound cofactor of specifier proteins involved in glucosinolate breakdown
Source: PLoS One. 2018 Nov 5;13(11):e0205755. doi: 10.1371/journal.pone.0205755 (PMC6218027; doi:10.1371/journal.pone.0205755)
Supplement: S2 Fig — Different amounts of TaTFP (in 37.5 mM sodium acetate buffer, pH 5, with 6.25% (w/v) sucrose) were denatured and precipitated. (A) Fe2+ in the supernatant was quantified colorimetrically using Ferene S reagent and calibration with (NH4)2Fe(SO4)2. (B) Iron in the supernatant was quantified by ICP-MS based on calibration with Iron ICP Standard. Each panel shows the results of three independent expression experiments. (PDF) [file pone.0205755.s002.pdf]

Iron is a centrally bound cofactor of specifier proteins involved in glucosinolate breakdown

Anita Backenköhler, Daniela Eisenschmidt, Nicola Schneegans, Matthias Strieker, Wolfgang Brandt, and Ute Wittstock

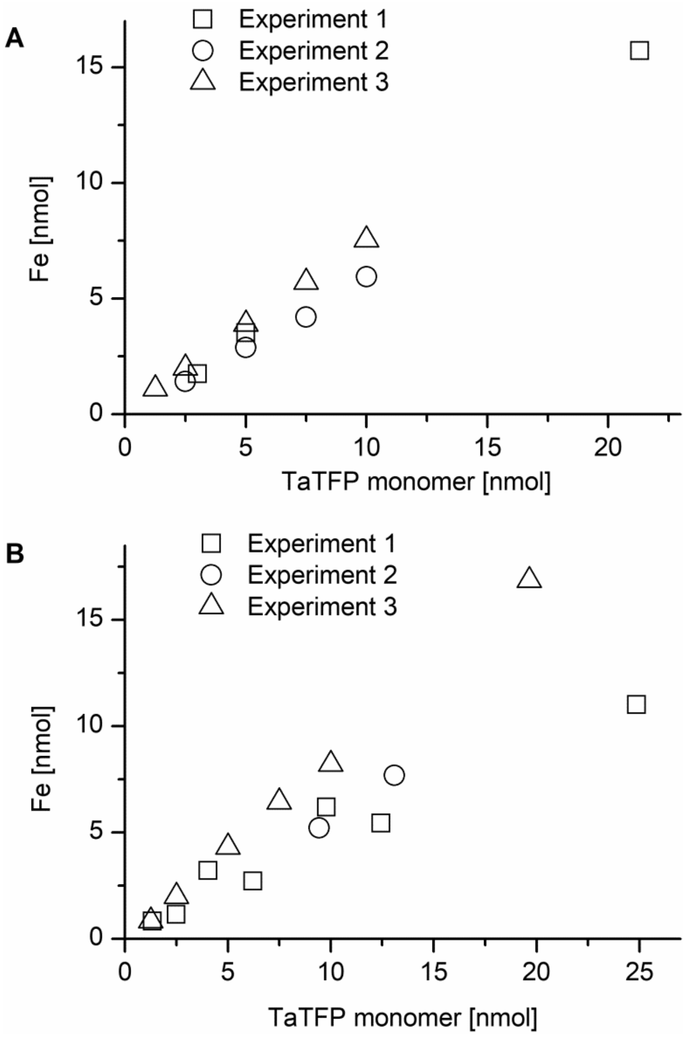

S2 Fig. Linearity of iron determinations of TaTFP. Different amounts of TaTFP (in 37.5 mM sodium acetate buffer, pH 5, with 6.25 % (w/v) sucrose) were denatured and precipitated. (A) Fe^{2+} in the supernatant was quantified colorimetrically using Ferene S reagent and calibration with $(NH_4)_2Fe(SO_4)_2$. (B) Iron in the supernatant was quantified by ICP-MS based on calibration with Iron ICP Standard. Each panel shows the results of three independent expression experiments.
