# Supplementary material for: Iron is a centrally bound cofactor of specifier proteins involved in glucosinolate breakdown
Source: PLoS One. 2018 Nov 5;13(11):e0205755. doi: 10.1371/journal.pone.0205755 (PMC6218027; doi:10.1371/journal.pone.0205755)
Supplement: S3 Fig — Purified TaTFP was incubated with allylglucosinolate and myrosinase in 50 mM MES buffer, pH 6.0, with or without 0.01 mM Fe2+ (FeII, (NH4)2Fe(SO4)2) or 0.01 mM Fe3+ (FeIII, NH4Fe(SO4)2) for 40 min. The amount of breakdown products is given in nmol per reaction. Shown are means ± SD of N = 5 independent expression experiments. The p value is given for a pairwise comparison of total amounts of product (Mann-Whitney test as errors were found to be non-normal). (PDF) [file pone.0205755.s003.pdf]

Iron is a centrally bound cofactor of specifier proteins involved in glucosinolate breakdown

Anita Backenköhler, Daniela Eisenschmidt, Nicola Schneegans, Matthias Strieker, Wolfgang Brandt, and Ute Wittstock

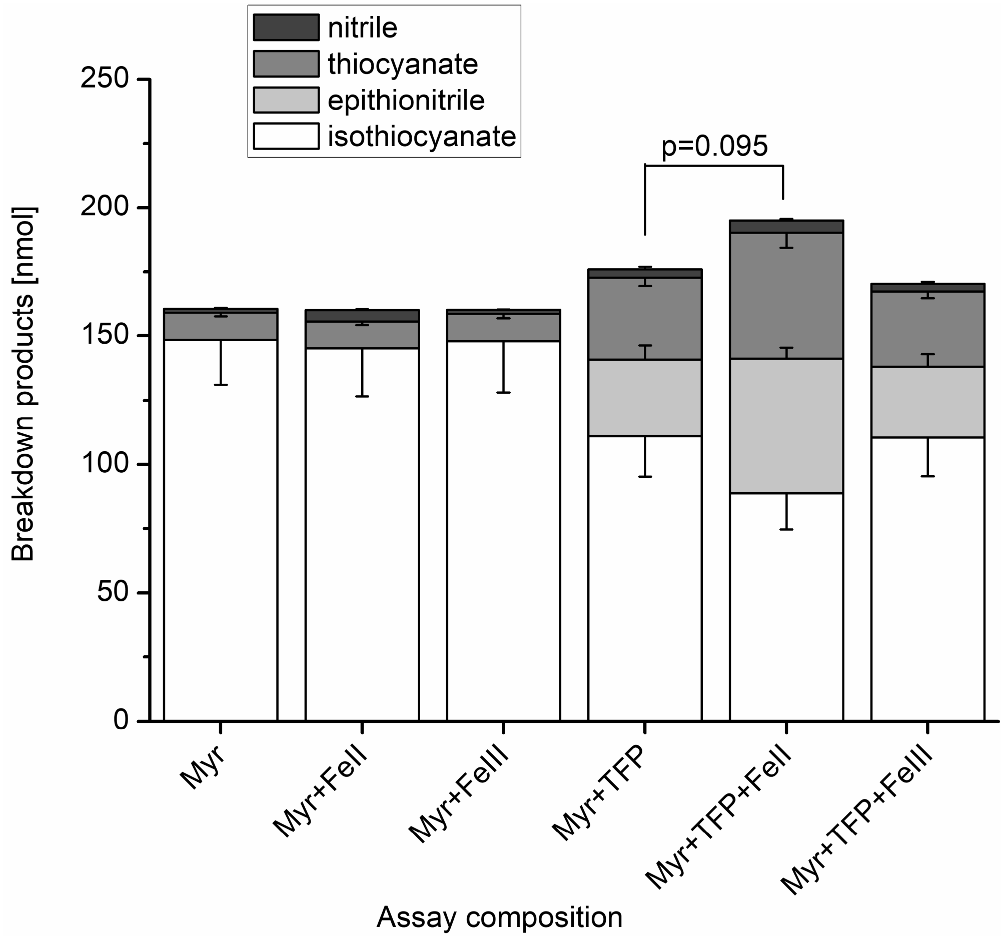

S3 Fig. Impact of Fe^{2+} and Fe^{3+} on product formation by myrosinase and TaTFP. Purified TaTFP was incubated with allylglucosinolate and myrosinase in 50 mM MES buffer, pH 6.0, with or without 0.01 mM Fe^{2+} (FeII, $(NH_4)_2Fe(SO_4)_2$) or 0.01 mM Fe^{3+} (FeIII, $NH_4Fe(SO_4)_2$) for 40 min. The amount of breakdown products is given in nmol per reaction. Shown are means ± SD of N=5 independent expression experiments. The p value is given for a pairwise comparison of total amounts of product (Mann-Whitney test as errors were found to be non-normal).
