# Supplementary material for: Iron is a centrally bound cofactor of specifier proteins involved in glucosinolate breakdown
Source: PLoS One. 2018 Nov 5;13(11):e0205755. doi: 10.1371/journal.pone.0205755 (PMC6218027; doi:10.1371/journal.pone.0205755)
Supplement: S4 Fig — Positions shown as active site residues of AtNSP3 in S5 Fig are highlighted with blue background if identical among all three sequences and with cyan background if identical in only two sequences. The proposed Fe2+-binding triad is marked with asterisks above the alignment. Among the other positions, identity of the three sequences is indicated by black background, amino acid similarity by gray background. Amino acid sequences were aligned using ClustalW (https://embnet.vital-it.ch/software/ClustalW.html). Shading was introduced by Boxshade (https://embnet.vital-it.ch/software/BOX_form.html) and color-edited manually. (PDF) [file pone.0205755.s004.pdf]

Iron is a centrally bound cofactor of specifier proteins involved in glucosinolate breakdown

Anita Backenköhler, Daniela Eisenschmidt, Nicola Schneegans, Matthias Strieker, Wolfgang Brandt, and Ute Wittstock

```
TaTFP        1 ------------------------------------------------------------
AtNSP3       1 MAQKLVAQGGETGDVWDDGVYDNVTKVYVGQGQYGIAFVKFEYANGSEVVVGDEHGEKTE
AtNSP1       1 MAQKLEAKGGEMGDVWDDGVYENVRKVYVGQAQYGIAFVKFEYVNGSQVVVGDEHGKKTE

TaTFP        1 ------------------------------------------------------------
AtNSP3      61 LGVEEFEIDSDDYIVYVEGYREKVSDMTSEMITFLSFKTSKGKTSQPIVKKPGVKFVLHG
AtNSP1      61 LGVEEFEIDADDYIVYVEGYREKVNDMTSEMITFLSIKTFKGKTSHPIEKRPGVKFVLHG

TaTFP        1 ------------------------MARTLQGEWMKVEQKGGQVPAPRSSHGIAVIGDKLY
AtNSP3     121 GKIVGFHGRSTDVLHSLGAYVSLPSTPKLLGNWIKVEQNG-EGPGLRCSHGIAQVGNKIY
AtNSP1     121 GKIVGFHGRSTDVLHSLGAYVSLSSTIKLLGKWIKVEQKG-EGPGLRCSHGIAQVGNKIY

TaTFP       37 CFGGEDPPYESIDNDLYVFDFNTHTWSIAPANGDVPKTRVLGTRMVAVGTKLYVFGGRNK
AtNSP3     180 SFGGELIPNQPIDKHLYVFDLETRTWSIAPATGDVPHLSCLGVRMVSVGSTLYTFGGRDF
AtNSP1     180 SFGGEFTPNQPIDKHLYVFDLETRTWSISPATGDVPHLSCLGVRMVSVGSTLYVFGGRDA

TaTFP       97 QLEFEDFYSYDTVKEEWKFLTKLDEKGGPEARTFHSMTSDENHVYVFGGVSKGGLNATPF
AtNSP3     240 SRQYNGFYSFDTTTNEWKLLTPVEE--GPTPRSFHSMAADEENVYVFGGVG------AMD
AtNSP1     240 SRQYNGFYSFDTTTNEWKLLTPVEE--GPTPRSFHSMAADEENVYVFGGVS------ATA

TaTFP      157 RFRTIEAYNIAEGKWAQLPDPGEDFEKRGMAGFLVVQGKLWVFYGFATANDPKIPTLYGS
AtNSP3     292 RIKTLDSYNIVDKTWFHCSNPGDSFSIRGGAGLEVVQGKVWIVYGFN-------------
AtNSP1     292 RLNTLDSYNIVDKKWFHCSTPGDSLTARGGAGLEVVQGKVWVVYGFN-------------

                                                                *   *   *
TaTFP      217 QDYESNRVHCYDPATQKWTEVETTGFEKPSRRSCFAHAAVGKYIIIFGGEIERDPEAHQG
AtNSP3     339 -GCEVDDVHFYDPAEDKWTQVETFGVK-PNERSVFASAAIGKHIVIFGGEIAMDPRAHVG
AtNSP1     339 -GCEVDDVHYYDPVQDKWTQVETFGVR-PSERSVFASAAIGKHIVIFGGEIAMDPLAHVG

TaTFP      277 PGTLSREGFALDTETLVWERYEGGP---IKPSNRGWVASTTTTINGKKGLLVHGGKLMTN
AtNSP3     397 PGQLIDGTFALDTETLQWERLDKFEG---TPSSRGWTASTTGTIDGKKGLVMHGGKAPTN
AtNSP1     397 PGQLTDGTFALDTETLQWERLDKFGGEEETPSSRGWTASTTATIDGKKGLVMHGGKAPTN

TaTFP      334 ERTDEMYFFAVNSST
AtNSP3     454 DRFDDLFFYGIDSV-
AtNSP1     457 DRFDDLFFYGIDSA-
```

S4 Fig. Amino acid sequence identity among TaTFP, AtNSP3, and AtNSP1. Positions shown as active site residues of AtNSP3 in S5 Fig are highlighted with blue background if

identical among all three sequences and with cyan background if identical in only two sequences. The proposed Fe^{2+}-binding triad is marked with asterisks above the alignment. Among the other positions, identity of the three sequences is indicated by black background, amino acid similarity by gray background. Amino acid sequences were aligned using ClustalW (https://embnet.vital-it.ch/software/ClustalW.html). Shading was introduced by Boxshade (https://embnet.vital-it.ch/software/BOX_form.html) and color-edited manually.
