# Supplementary material for: Iron is a centrally bound cofactor of specifier proteins involved in glucosinolate breakdown
Source: PLoS One. 2018 Nov 5;13(11):e0205755. doi: 10.1371/journal.pone.0205755 (PMC6218027; doi:10.1371/journal.pone.0205755)
Supplement: S5 Fig — (A) Model of the AtNSP3 active site with docked Fe2+ and allylglucosinolate aglucone. (B) AtNSP1 active site as represented in the crystal structure (PDB 5GQT, [36]). (C) TaTFP active site with docked Fe2+ and allylglucosinolate aglucone [34] derived from the crystal structure (PDB 5A10). An alignment of the three amino acid sequences is provided in S4 Fig. (PDF) [file pone.0205755.s005.pdf]

**Iron is a centrally bound cofactor of specifier proteins involved in glucosinolate breakdown**

Anita Backenköhler, Daniela Eisenschmidt, Nicola Schneegans, Matthias Strieker, Wolfgang Brandt, and Ute Wittstock

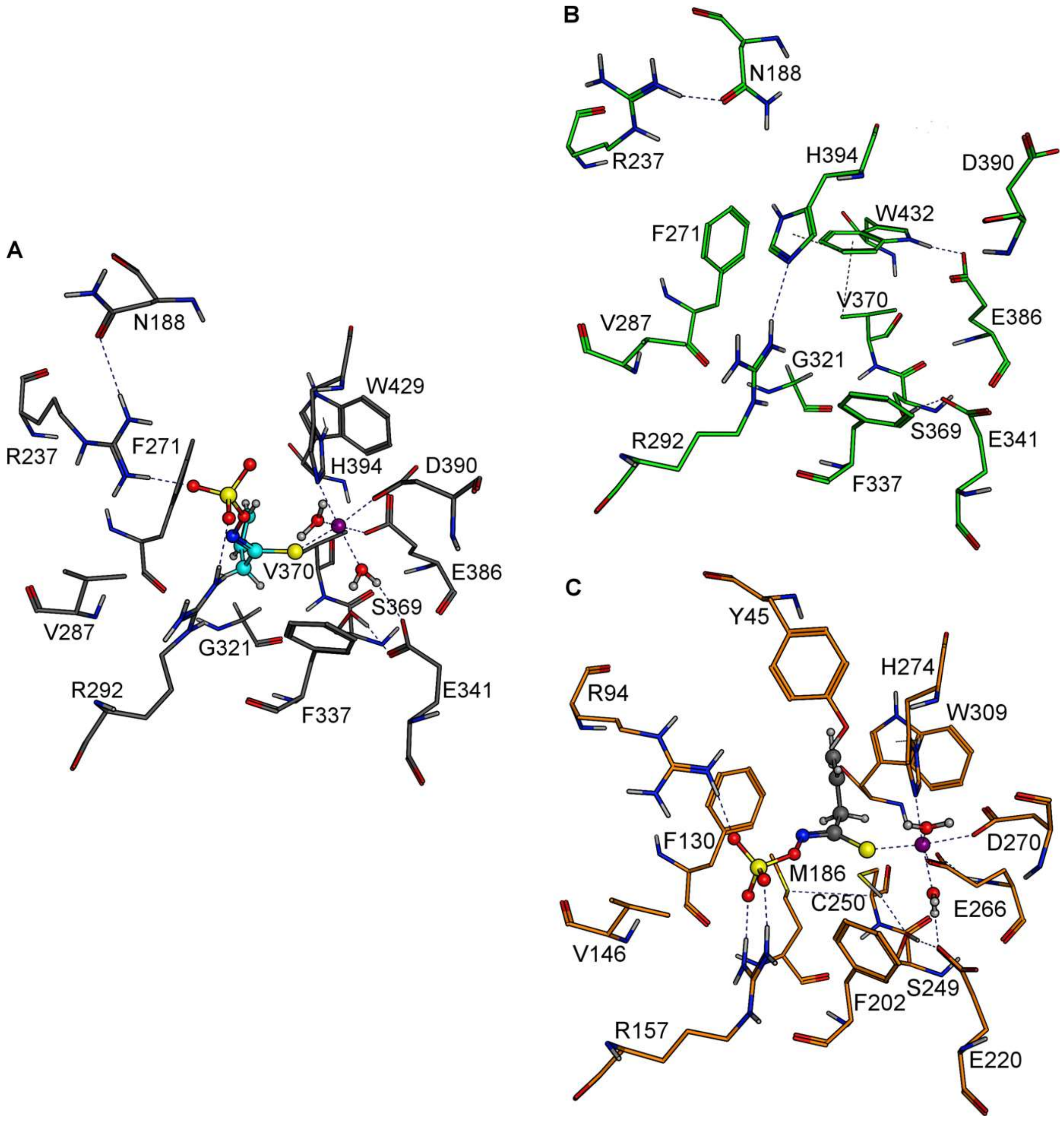


**S5 Fig. Active site of AtNSP3 in comparison with those of TaTFP and AtNSP1.** (A) Model of the AtNSP3 active site with docked $Fe^{2+}$ and allylglucosinolate aglucone. (B) AtNSP1 active site as represented in the crystal structure (PDB 5GQT, [36]). (C) TaTFP

active site with docked $Fe^{2+}$ and allylglucosinolate aglucone [34] derived from the crystal structure (PDB 5A10). An alignment of the three amino acid sequences is provided in S4 Fig.
