# Supplementary material for: Iron is a centrally bound cofactor of specifier proteins involved in glucosinolate breakdown
Source: PLoS One. 2018 Nov 5;13(11):e0205755. doi: 10.1371/journal.pone.0205755 (PMC6218027; doi:10.1371/journal.pone.0205755)
Supplement: S1 Table — Main chain parameters comprises the overall Ramachandran plot quality, peptide bond planarity, hydrogen bond energy, bad non-bonded interactions, and G-factor. Side chain parameters comprises chi1 and chi2 dihedrals. (PDF) [file pone.0205755.s006.pdf]

Iron is a centrally bound cofactor of specifier proteins involved in glucosinolate breakdown

Anita Backenköhler, Daniela Eisenschmidt, Nicola Schneegans, Matthias Strieker, Wolfgang Brandt, and Ute Wittstock

S1 Table. Model quality of AtNSP3 evaluated with PROCHECK. Main chain parameters comprises the overall Ramachandran plot quality, peptide bond planarity, hydrogen bond energy, bad non-bonded interactions, and G-factor. Side chain parameters comprises chi1 and chi2 dihedrals.

PROCHECK parameter	Evaluation result
Ramachandran Plot	86.8 % most favored<br>12.4 % additionally allowed<br>0.5 % generally allowed<br>0.3 % disallowed
Main chain parameters	5 better, 1 inside, 0 worse
Side chain parameters	5 better, 0 insider, 0 worse
Main chain Bond lengths	100.0 % within limits
Main chain bond angles	92.6 % within limits, 7.4 % highlighted
Planar groups	91.3 % within limits, 8.7 % highlighted
