# Supplementary material for: Iron is a centrally bound cofactor of specifier proteins involved in glucosinolate breakdown
Source: PLoS One. 2018 Nov 5;13(11):e0205755. doi: 10.1371/journal.pone.0205755 (PMC6218027; doi:10.1371/journal.pone.0205755)
Supplement: S2 Table — The ten poses with lowest AtNSP3-aglucone interaction energies are included. Fulfillment of the three essential docking criteria is indicated. (PDF) [file pone.0205755.s007.pdf]

Iron is a centrally bound cofactor of specifier proteins involved in glucosinolate breakdown

Anita Backenköhler, Daniela Eisenschmidt, Nicola Schneegans, Matthias Strieker, Wolfgang Brandt, and Ute Wittstock

S2 Table. Evaluation of GOLD docking poses obtained with AtNSP3, Fe^{2+} and allylglucosinolate aglucone. The ten poses with lowest AtNSP3-aglucone interaction energies are included. Fulfillment of the three essential docking criteria is indicated.

Pose	Interaction energy ($kJ\ mol^{-1}$)	Aglucone-Fe^{2+} coordination	Sulfate group stabilization	Side chain interaction
1	-473.63	thiolate (S^-)	-	H394, F271
2	-463.17	thiolate (S^-)	-	H394, F271
3	-348.11	thiolate (S^-)	R237	-
4	-340.99	thiolate (S^-)	R237	-
5	-340.99	thiolate (S^-)	R237	-
6	-338.49	thiolate (S^-)	R237, R292	-
7	-336.39	sulfate	R237	-
8	-335.56	thiolate (S^-)	R237, R292	H394, F271
9	-333.05	thiolate (S^-)	R237, R292	H394, F271
10	-333.05	thiolate (S^-)	R237	F271
